# Sex‐related patterns in the electroencephalogram and their relevance in machine learning classifiers

**DOI:** 10.1002/hbm.26417

**Published:** 2023-07-17

**Authors:** Thomas Jochmann, Marc S. Seibel, Elisabeth Jochmann, Sheraz Khan, Matti S. Hämäläinen, Jens Haueisen

**Affiliations:** ^1^ Department of Computer Science and Automation Technische Universität Ilmenau Ilmenau Germany; ^2^ Department of Neurology Jena University Hospital Jena Germany; ^3^ Athinoula A. Martinos Center for Biomedical Imaging Massachusetts General Hospital Charlestown Massachusetts USA; ^4^ Harvard Medical School Boston Massachusetts USA; ^5^ Department of Neuroscience and Biomedical Engineering, School of Science Aalto University Espoo Finland

**Keywords:** artificial intelligence, deep learning, electroencephalography, explainable artificial intelligence, machine learning, sex differences

## Abstract

Deep learning is increasingly being proposed for detecting neurological and psychiatric diseases from electroencephalogram (EEG) data but the method is prone to inadvertently incorporate biases from training data and exploit illegitimate patterns. The recent demonstration that deep learning can detect the sex from EEG implies potential sex‐related biases in deep learning‐based disease detectors for the many diseases with unequal prevalence between males and females. In this work, we present the male‐ and female‐typical patterns used by a convolutional neural network that detects the sex from clinical EEG (81% accuracy in a separate test set with 142 patients). We considered neural sources, anatomical differences, and non‐neural artifacts as sources of differences in the EEG curves. Using EEGs from 1140 patients, we found electrocardiac artifacts to be leaking into the supposedly brain activity‐based classifiers. Nevertheless, the sex remained detectable after rejecting heart‐related and other artifacts. In the cleaned data, EEG topographies were critical to detect the sex, but waveforms and frequencies were not. None of the traditional frequency bands was particularly important for sex detection. We were able to determine the sex even from EEGs with shuffled time points and therewith completely destroyed waveforms. Researchers should consider neural and non‐neural sources as potential origins of sex differences in their data, they should maintain best practices of artifact rejection, even when datasets are large, and they should test their classifiers for sex biases.

## Graphical abstract

Deep learning is increasingly being proposed for detecting neurological and psychiatric diseases from electroencephalogram (EEG) data but the method is prone to inadvertently incorporate biases from training data and exploit illegitimate patterns. In this work, we derived the male‐ and female‐typical patterns used by a convolutional neural network that detects the sex from clinical EEG and we found electrocardiac artifacts to be leaking into the supposedly brain activity‐based classifiers. None of the traditional frequency bands was particularly important for sex detection and we were able to determine the sex even from EEGs with shuffled time points and therewith completely destroyed waveforms.

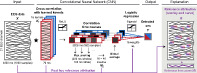

## 1 INTRODUCTION

The advent of deep learning has led to numerous studies about convolutional neural networks (CNNs) for detecting neurological and psychiatric diseases from electroencephalogram (EEG) data (Craik et al., 2019; Jonas et al., 2019; Roy et al., 2019; Schirrmeister et al., 2017). CNNs are intriguing for their potential to uncover and utilize previously unknown patterns from the EEG, as the distinctive patterns can be learned from the data and do not need to be handcrafted with prior assumptions. These patterns, however, are estimated only from their correlation with the outcome, and are not designed with biomedical rigor. CNNs and many other deep learning techniques can therefore be prone to exploiting illegitimate criteria (Kapoor & Narayanan, 2022) that are not directly related to the pathology itself, for example, the sex. Such sex‐biased patterns can be of neural and non‐neural origin. Either way, the distinctive features in many machine learning classifiers remain hidden (Heinrichs & Eickhoff, 2020), as explainability is often deemed unnecessary or the required explainability methods are unavailable for the used technique. Machine learning‐based studies typically involve larger datasets than conventional EEG studies. Often, the individual recordings from such large datasets are treated less thoroughly, as demonstrated by the number of studies reviewed by Craik et al. (2019) that leave artifacts in during analysis. These studies include artifacts, potentially sex‐biased, that are less present in conventional studies.

Many diseases that have been addressed with deep learning‐based EEG analyses (Roy et al., 2019) have a pronounced unequal prevalence between males and females. For example, major depressive disorder and Alzheimer disease are more prevalent in females, whereas substance use disorders and Parkinson disease are more prevalent in males (Salminen et al., 2022). Long reported sex differences in the EEG (Brenner et al., 1995; Cave & Barry, 2021; Clarke et al., 2001; Davidson et al., 1976; Friedl & Vogel, 1979a, 1979b; Jaušovec & Jaušovec, 2010; Maciejewska & Froelich, 2021; Smith, 1952; Veldhuizen et al., 1993; Vogel & Götze, 1962; Wada et al., 1994) and automatic sex‐detection with machine learning (Kaushik et al., 2018; Maciejewska & Froelich, 2021; van Putten et al., 2018) imply that sex‐related patterns can be a confounder to machine learning‐based assessments from EEG. The machine learning models might involve the patient's sex in predictions that should only be based on pathology. Sex‐related biases in EEG datasets can occur from unequal prevalence of diseases and actual electrophysiological sex differences but also from unequal diagnosing or unequal willingness to seek treatment.

In this work, we address three main research questions:

1. *Does it require special network architectures to detect the sex from EEG or is sex likely detectable by the commonly used CNN architectures?*
2. *Which patterns in the EEG signal are relevant to the CNN for detecting the sex and what do these patterns look like?*
3. *Can we isolate neural and non‐neural sources of the sex differences in the EEG?*

We demonstrate the influence of non‐neural artifacts, particularly from the heart, on deep learning‐based sex detection and propose mitigation measures to reduce the confounding impact of such artifacts. On the artifact‐cleaned and, thus, supposedly more brain‐originated data, we present observations on topographies, waveforms, and frequencies, allowing for new hypotheses about the sources of sex differences in the EEG. Finally, we discuss the implications of the sex's detectability to machine learning‐based disease assessments.

## 2 MATERIALS AND METHODS

### 2.1 Dataset preparation and signal filtering

We conducted our study on the clinical TUH Abnormal EEG Corpus (2.0.0) (Lopez de Diego, 2017; Obeid & Picone, 2016), selecting all 1505 recordings that were annotated as *normal* and came from adult patients. Sixty‐seven recordings were removed for having a different sampling rate compared with the rest, in suspicion of bias‐inducing unknown differences. Only one recording per patient was allowed, leading to the removal of another 107 recordings. We selected the one recording per patient where the Autoreject algorithm (Jas et al., 2017; implemented in MNE Python; Gramfort et al., 2013) returned the most usable segments, which depended on the recording length and amount of noise. The recordings in the dataset were split into separate directories for training and evaluation by the dataset's authors and we kept that split in our work to allow comparisons to other studies (training subset: 1140 patients, 636 (56%) female, 504 (44%) male; evaluation subset: 142 other patients, 61 male (43%), 81 female (57%), age range 18–88 years, age mean ± SD = 45 ± 17 years). We aimed to utilize as much as possible of the available information and accounted for the imbalance in the corpus by using class weights for the calculation of the cost function during network training, by reporting balanced accuracies (see definition below) for reporting the results, and by deriving $p_{0}$ in the statistical tests from the population mean (57% females). Per recording (250 Hz sampling rate, 21 EEG channels in 10–20 configuration), 50 non‐overlapping segments (4 s length) were selected using the Autoreject method. Autoreject scanned through the data and returned segments with low noise and artifact load, regardless of the underlying status of the recording. The first 2 min of each recording were skipped, as readjustments of the electrodes often occur at the beginning, leading to an increased number of bad channels (Figure S1 shows the average artifact load over time). The optimal segment length was determined in a parameter study between 2 and 8 s that showed no benefit from using more than 4 s (Figure S2). Forty‐nine recordings that did not yield a sufficient number of segments due to noise and artifacts were excluded. We chose a threshold of 50 segments because larger numbers would have led to the exclusion of substantially more cases and smaller numbers would have left more data unused. The corpus was built from archival clinical EEG data reflecting a broad spectrum of clinical scenarios. From these heterogeneous data, the selection of the segments was purely based on signal quality (determined by Autoreject) and did not depend on the state of the patient or the experiment, that is, eyes could have been open or closed, patients could have received photic stimulation, undergo behavioral tasks, et cetera. The signals were bandpass filtered from 1 to 40 Hz to show that the observed effects occur within this commonly studied frequency range (Hari & Puce, 2017).

After interpolation of bad channels, artifacts were filtered with independent component analysis (ICA). The artifactual ECG and EOG components were semi‐automatically selected and removed using the Corrmap method (Campos Viola et al., 2009). That algorithm rejected on average two artifactual components out of a maximum of 20 ICA components. Finally, the segments were referenced to common average, de‐meaned, and normalized to unit standard deviation.

### 2.2 A simple CNN for sex detection

**FIGURE 1:**
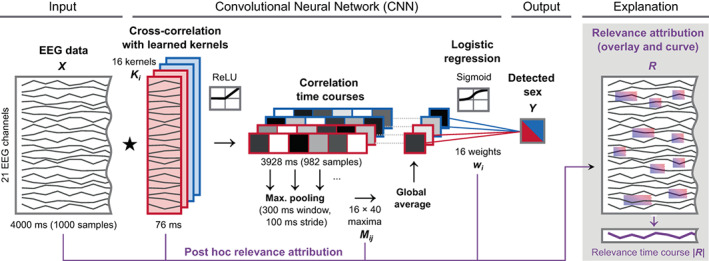
Sex detection with the *Mini‐SpaTeN* convolutional neural network and relevance attribution method. First, the segment $\left(X\right)$ is cross‐correlated $\left(⋆\right)$ with 16 learned spatio‐temporal kernels ($K_{i}$) similar in dimension as short windows of EEG (actual kernels depicted in Figure 2). Since the kernels have the same number of channels as the data, they only slide along the time axis and not across channels. The 16 correlation curves are rectified (ReLU activation) and divided into 40 overlapping windows. Next, the window‐wise maxima ($M_{\mathrm{ij}}$) are averaged. In the final layer, the sex $\left(Y\right)$ is predicted from these 16 averages with logistic regression. Post hoc, the network parameters are used to attribute the relevance R that each EEG channel and time point from a recording had to the prediction (path indicated in purple). The signs of the final classifier layer weights ($w_{i}$) imply the sex corresponding to each kernel from the first layer (−/female/red and +/male/blue).

We built a CNN that detects a patient's sex from few‐second segments of EEG. We designed an extremely small and shallow network architecture under the conjecture that findings from this architecture generalize to most deep learning architectures for EEG analysis; whatever this CNN can detect should easily be detected by deeper networks because of their higher fitting capacity (Goodfellow et al., 2016, pp. 423 ff.). Table 1 lists the full network architecture and hyperparameters, and Figure 1 illustrates the data flow in the network. The network was implemented in TensorFlow 2.7.0 (Abadi et al., 2016; Chollet, 2015) and was termed *Mini*malistic *Spa*tio‐*Te*mporal *N*etwork (Mini‐SpaTeN).

**TABLE 1:** Layers of our CNN architecture for sex detection. The two trainable layers are marked by cursive font.

	Layer type, specifications	Output shape	Comment
0.	Input EEG segment (4000 ms)	21 × 1000	1000samples≙4000ms
1.	*2D convolution* (16 kernels, 21 × 19, ReLU activation)	16 × 982	19samples≙76ms
2.	Maximum pooling (1 × 75, 1 × 25 stride)	16 × 40	75samples≙300ms
3.	Global average pooling	16	Average over time
4.	*Dense* (sigmoid activation)	1	Predicted sex (0, 1)

We designed the CNN with the practical minimum of only two trainable layers: one *convolution* layer and one *dense* layer. The network works as follows: The convolution layer receives the EEG data matrix as input and calculates the *cross‐correlation* between the EEG data matrix and filter matrices, denoted as kernels. The values of the kernels are trainable parameters, whereas the number and length of the kernels are non‐trainable hyperparameters. We tested the influence of these hyperparameters and found the accuracy of the sex detection to be similar for a wide range of kernel numbers and lengths (Figure S3). We then chose values that are suitable for visual inspection and printing of the learned parameters (16 kernels, 19 samples length, equivalent to 76 ms). The kernels are short, EEG‐like patterns. These spatio‐temporal kernels can reflect topographies, waveforms, and frequencies, as well as cross‐spatial, cross‐temporal, and cross‐spectral relations. Arbitrary waveforms can be learned, addressing the importance of non‐sinusoidal waveforms (Cole & Voytek, 2017; Jasper, 1948). The cross‐correlation of the kernels with the EEG segment leads to one time course per kernel, representing how each of the kernels matches with the EEG data over time. Next, an unbiased *ReLU* (rectifying linear unit; Glorot et al., 2011) activation function is applied to these time courses, which sets all negative correlation values to zero and leaves positive values as they are. For each of the time courses, the network then calculates a single value that is high, when the data contain segments that are particularly similar to a kernel. Instead of using only a single maximally correlating value per kernel, we averaged the maximum correlation values over 40 overlapping intervals (via a maximum pooling layer and a global averaging layer) to increase the robustness. Finally, from the 16 averages, the dense layer with a sigmoid activation function classifies the sex, identical to logistic regression. We chose a sigmoid activation in the output layer to map the output to a range between 0 and 1, corresponding to the encoded sex (0 = female, 1 = male). Since the ReLU function in the first layer crops the negative values, the sign of the weights in the logistic regression layer directly tells which kernel is male‐ and which is female‐typical.

We trained the CNN to minimize the binary cross‐entropy loss of the accuracy, balanced with class weights, via adaptive moment estimation backpropagation, *Adam* (Kingma & Ba, 2017). Convergence of the loss metric was typically reached after 5 epochs (an epoch denotes one iteration over the full dataset). We kept iterating for a total of 20 epochs and selected the model with the best performance on a 20% held‐out portion, neither used for training the weights nor for later evaluation. The weights of the kernels and the logistic regression layer are randomly initialized and during the training process, they iteratively converge toward patterns/values that are optimal for the classification task (in this case sex detection).

### 2.3 Predictions with the trained neural network and performance evaluation

Per‐subject sex predictions are made from a majority vote after applying the network to 50 separate segments. We report balanced accuracies, defined as the arithmetic mean of true positive rate and true negative rate (Brodersen et al., 2010), to account for the imbalanced number of males and females. For better comparison with other studies, we also report conventional accuracies as the percentage of correct predictions. Values are reported with their means and standard deviations from 30 networks that were independently randomly initialized and trained on the same data. To probe the accuracy limit of this network architecture, we increased the number of kernels to 512 and made an ensemble prediction by a majority vote from 30 networks, again, independently randomly initialized and trained on the same data.

We used a one‐sided binomial test with Bonferroni correction for n=15 multiple comparisons resulting in an adjusted significance level of α=0.003. $p_{0}$ was derived from the population mean (142 subjects, 57% female).

### 2.4 Feature visualization and relevance attribution

Our shallow network architecture brought the advantage that the distinctive patterns could be directly read from the learned weights. The learned weights consist of the elements of the convolutional kernels and of the dense output layer. The network, up to the next‐to‐last layer, yields how well each kernel, $K_{k}$, matches with the data. Then, the sign of the weights, $W_{k}$, in the dense output layer tells whether matching with an individual kernel turns the prediction toward male or female.

We determined the *relevance* R of the EEG signal at each channel and time point for the network's decision between male and female. The shallowness and simplicity of the network allowed for a short derivation of R without involving an extensive explainability framework: The CNN scans through each 4‐s EEG segment, X, in 40 overlapping windows, $X_{i}$, of 300 ms length and 100 ms overlap. Within these windows, only the parts with the maximum correlation between the data and the kernels contribute to the final prediction. The scanning first leads to the cross‐correlation time courses $C_{k}$ between the data matrices and a kernels

$$C_{i,k}=X_{i}⋆K_{k}.$$

To calculate the relevance matrices $R_{i,k}$ at the maximum correlation time points in each window from each kernel, we first defined

$$R_{i,k}^{\prime }=\left(X\left(\arg\;\max\left(C_{i,k}\right)\right)⊙K_{k}\right)\cdot \max\left(C_{i,k}\right),$$

where $X\left(\arg\;\max\left(C_{i,k}\right)\right)$ is a submatrix of the same size as the kernels, and ⊙ denotes the element‐wise Hadamard product. Next,

$$R_{i,k}=\mathrm{sign}\left(R_{i,k}^{\prime }\right)\cdot \sqrt{|R_{i,k}^{\prime }|}\cdot w_{k}.$$

We then summed up the relevances from the different segments at their corresponding time points, which yields kernel‐specific relevance matrices with the same dimensions as the data matrices. Finally, the sum over all kernel‐specific relevance matrices was used to determine the total relevance that we then overlayed in a red and blue tint on top of the data (Figure 1).

The sum of a relevance matrix over all channels yields a relevance time course. We used these relevance time courses to assess the impact of electrocardiac artifacts on the CNN's predictions, by comparing averaged time courses of the absolute relevance ∣R∣ with averaged time courses of the ECG around the QRS complexes.

### 2.5 Explorative data modification

To assess the influence of artifacts, we compared the accuracies and relevances with and without ICA filtering. On the ICA‐filtered data, we tested different preprocessing on the entire dataset (training *and* evaluation data) to probe the origin of the sex differences in the EEG:

- We tested if we could isolate the origin of the sex difference to one of the traditional frequency bands by bandpass filtering.
- To assess the importance of the *time domain*, we randomly shuffled the time points of the EEG signals within each segment, destroying waveforms and cross‐temporal connectivity. We repeated this on band‐filtered data in the traditional EEG bands. These experiments required altering the network architecture to only learn topographic kernels (i.e. EEG patterns from a single time point).
- To assess the importance of the *spatial domain*, we destroyed the topographies: we randomly moved each channel in each segment up to 200 ms forward or backward (in the data before the whole training and analysis procedure). This kept the waveforms and frequencies within each channel intact.
- We computed the accuracy of detecting the sex directly from a single ECG channel instead of from the EEG.

### 2.6 Sex or gender

We use the patient sex from the metainformation of the EEG data, encoded as 0 or 1, as the target of our neural network. Throughout this work, we discuss *sex* rather than *gender* because the dataset was built from clinical data during a time (2002–2013) when patients presumably presented with the sex information related to their legal documents, at that time containing the sex assigned at birth rather than their self‐identified gender.

## 3 RESULTS

### 3.1 Detectability of the patients' sex from raw, filtered, and experimentally modified data

Table 2 lists the accuracies from the different experiments. On EEG data that had not received any artifact treatment, other than automatically selecting segments with low artifact load, our CNN detected the patients' sex with 78% ± 2% accuracy (p<.001, balanced accuracy, majority vote over 50 segments per patient, accuracy from single segments was 73% ± 1%). After artifact removal, the balanced accuracy dropped to 74% ± 2% $\left(p<.001\right)$. Repeating the entire pipeline (training and prediction) with band‐filtered data (δ,θ,α,β,γ; frequency ranges listed in Table 2) resulted in the same or slightly smaller accuracies. None of the frequency bands stood out and the largest difference between any two of the frequency bands was 4% (between α and γ). The *p*‐values from pairwise significance tests between the frequency bands are provided in Table S1. When shuffling the time domain and shrinking the spatio‐temporal kernels to a single time point, which only represents a topography rather than a topographic temporal evolution, the detection was still possible (68% ± 3%, p=.002). The detection from topographies was also possible from band‐filtered data in each of the frequency bands and even lead to higher accuracies (up to 5% increased). Again, none of the frequency bands stood out. The largest difference between any two of the frequency bands was only 3% (between α and γ). The *p*‐values from pairwise significance tests between the frequency bands are provided in Table S2. With spatio‐temporal kernels and shuffled topographies, the detection was no longer possible (61%, p=.17). We estimated a balanced accuracy of 81% for this architecture and dataset with the ensemble prediction from 30 networks with an increased number of kernels (512).

**TABLE 2:** Sex detection accuracies of per‐subject predictions from majority votes over 50 segments (mean ± SD over 30 randomly initialized networks).

Experiment^a^	Kernels^b^	Accuracy		*p* ^c^	*p* ^d^
		Balanced	Imbalanced	α/n=0.003	α/n=0.01
*EEG*					
Raw, 1–40 Hz	21 × 19	78%±2%	78%±2%	<.001	
ICA, 1–40 Hz	21 × 19	74%±2%	75%±2%	<.001	
1–4 Hz $\left(\delta \right)$	21 × 19	73%±2%	74%±2%	<.001	
4–8 Hz $\left(\theta \right)$	21 × 19	73%±3%	74%±3%	<.001	
8–14 Hz $\left(\alpha \right)$	21 × 19	74%±2%	75%±2%	<.001	
14–30 Hz $\left(\beta \right)$	21 × 19	71%±3%	72%±2%	<.001	
30–40 Hz $\left(\gamma \right)$	21 × 19	70%±3%	70%±2%	<.001	
ICA, 1–40 Hz, shuffled time points	21 × 1	68%±3%	69%±2%	.002	↰
1–4 Hz $\left(\delta \right)$	21 × 1	72%±2%	73%±2%	<.001	<.001
4–8 Hz $\left(\theta \right)$	21 × 1	71%±2%	71%±2%	<.001	0.007
8–14 Hz $\left(\alpha \right)$	21 × 1	73%±2%	73%±2%	<.001	<.001
14–30 Hz $\left(\beta \right)$	21 × 1	72%±2%	73%±2%	<.001	<.001
30–40 Hz $\left(\gamma \right)$	21 × 1	70%±2%	70%±2%	<.001	0.11
ICA, 1–40 Hz random channel phase^e^	21 × 19	61%±3%	61%±3%	.17	
**Raw, 1–40 Hz, ensemble**	21 × 19	**81%**	82%	<.001	
*ECG*					
Raw, 1–40 Hz	1 × 19	58%±2%	60%±1%	.22	

*Note*: To probe the accuracy limit of this network architecture, we increased the number of kernels from 16 to 512 and made an ensemble prediction by a majority vote from 30 independently randomly initialized networks, trained on the same data (bold).

^a^ Filters and modifications were applied to training and evaluation data.

^b^ Size of the convolutional kernels in the CNN; channels × samples; $19\;\text{samples}\;\hat{=}\;76\;\mathrm{ms}$.

^c^ Binomial test on the (imbalanced) accuracy; 142 subjects, 57% female; Bonferroni adjusted alpha level of 0.003 per test (0.05/15) for n=15 multiple comparisons.

^d^ One‐sided paired *t*‐test on the (imbalanced) accuracy to compare the accuracies from the physiological frequency bands against the 1–40 Hz data; 142 subjects, 57% female.

^e^ Without fixed temporal relations between channels it is harder to populate multiple channels of one kernel. To neutralize this drawback, we increased the number of kernels for this experiment from 16 to 512.

We searched for commonalities between the falsely classified patients, but errors were found for all kinds of patients: males and females, young and old, with and without medication, and with diverse medical histories. The confusion matrix for the experiment with ICA‐filtered data and spatio‐temporal kernels (Table 3) showed no significant bias (p=.20).

**TABLE 3:** Confusion matrix for sex classification from EEG with ICA‐based artifact removal, filtered 1–40 Hz.

N=142	Estimated female	Estimated male	Total
Labeled female	67	14	81
Labeled male	22	39	61
Total	89	53	

*Note*: The values show no significant bias (p=.20, two‐sided binomial test).

A single‐channel adaptation of our shallow CNN could not predict the sex from an ECG channel (58%, p=.22). For single EEG channels, the frontal channels reached the highest accuracies When allowing more and more channels and always adding the optimal next channel, the selected sensors spread across the head. Six channels (F4, Fp2, C4, T6, Pz, O1) already led to an accuracy >70%. Figure 3 shows the accuracies from single EEG channels and the estimated order of optimal addition when always searching for the channel that leads to the largest increase in accuracy.

### 3.2 Relevance attribution with raw data compared with ICA‐filtered data

Figure 4 shows one typical segment from the evaluation data with a relevance map overlay. In the case of raw data, the visual assay showed a strong accumulation of relevance during the electrocardiac QRS complexes, but not during the intervals where T waves are seen in the ECG or when pulse waves normally occur. ICA‐based artifact removal strongly mitigated the accumulation of relevance. As illustrated in Figure 5, accumulation and mitigation were generally confirmed by averaging the relevance time courses around the QRS epochs from all evaluation subjects.

## 4 DISCUSSION

We predicted the sex from EEG with a shallow, explainable CNN, reproducing the recent results from a vastly deeper CNN by van Putten et al. (2018). With our relevance attribution technique and by exploratively altering the signals, we narrowed down potential neural and non‐neural sources of sex differences.

**FIGURE 2:**
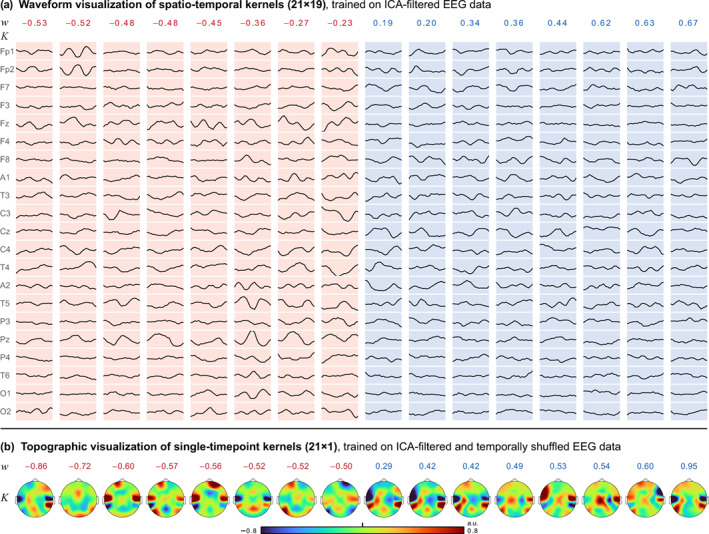
Learned male and female weights extracted from the trained CNN. (a) From the 16 weights w of the final classification layer, half individually converged to a positive and half to a negative sign, which determines the sex, related to the corresponding kernels below. Female weights are marked red, male weights are blue. The spatio‐temporal kernels K are similar in dimension to short segments of EEG, but may not be interpreted as EEG (Haufe et al., 2014). As the CNN searches for strong cross‐correlations between the kernels and the data, the kernel values can have three meanings: large kernel values with the same sign as the data are positively added to the correlation sum, small kernel values ignore the underlying data, and inverse values reduce the correlation sum and therewith the relevance of the whole segment. Thus, the kernel waveforms can also show *anti‐patterns*, instead of the searched distinctive activity. Part of a kernel can be concurrently correlated with sex differences and just be used for localization in the data, but not be sex‐different itself. (b) Weights w and single‐timepoint kernels K, trained on temporally shuffled data. Figure S4 shows a variant of (a) in which the spatio‐temporal kernels were trained on raw data instead of ICA‐filtered data. They show waveforms that more strongly resemble the cardiac QRS complex.

To explain the CNN and its predictions, we visualized the learned parameters (convolutional kernels and weights of the final classification layer) and estimated the relevance of the data points for a prediction from each segment. We refrain from neural interpretations of the spectra and the waveforms in the kernels (Figure 2) as they do not necessarily show neural activity (Haufe et al., 2014). The role of the different parts of the kernels during the convolution operation is to yield the maximum correlations at the relevant positions. This can be achieved by *recognition*: positively correlating with wanted patterns in the data; but also by *exclusion*: anti‐correlating with unwanted waveforms. Parts of a kernel can be used to steer the network toward intervals with sex differences, yet these parts themselves might not exhibit sex differences. Non‐neural differences also vary between EEG channels and should therefore not be mistaken for differences in the underlying brain regions.

**FIGURE 3:**
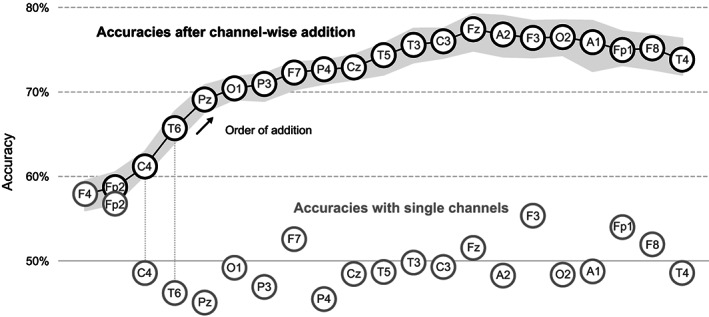
Accuracies from using only single channels and after adding channel by channel. The gray circles show the accuracies that are achieved when only a single channel is used. The chain of black circles shows the accuracy after step‐wise adding the channel that leads to the largest increase in accuracy. The gray envelope around the black chain indicates the standard deviation. F4 had the best single‐channel accuracy and, consequently, was the first in the chain. The lateral counterparts of the early members of the chain, for example, F3 and Fp1, were added only late to the chain, presumably due to redundant information.

**FIGURE 4:**
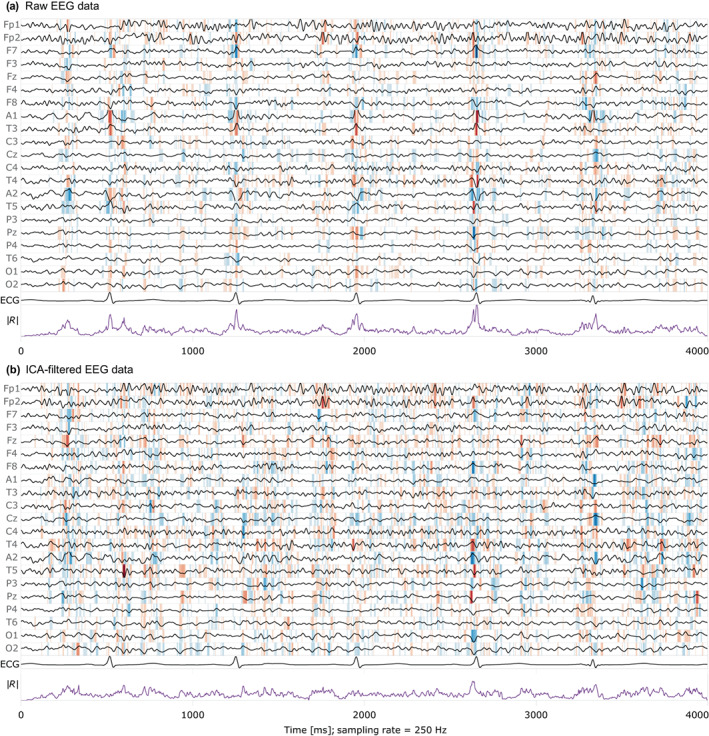
EEG signals with relevance visualization, ECG signal, and relevance time course from a typical example, with and without artifact removal (female, 45 years). Red background color indicates the influence of the data points toward predicting female, and blue toward male sex. The stronger the color, the larger the relevance. The last curve (purple) shows the absolute relevance ∣R∣, summarized over all EEG channels. Second to last is the ECG channel. With raw data (a), relevance accumulates at the times of the QRS complexes. After ICA‐based artifact removal (b), the relevance is more evenly distributed over the data, and therewith non‐cardiac signals are factored in into the decision more strongly.

**FIGURE 5:**
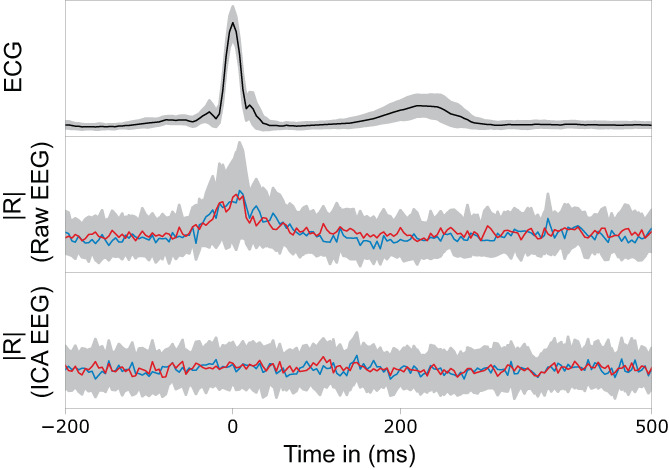
Comparison of the ECG signal with the total relevance, averaged over all evaluation subjects, with and without ICA‐filtering. The relevance of the raw EEG is increased around the QRS complexes, but not during the average T wave and pulse wave epochs (middle). For ICA‐filtered EEG, the relevance is evenly distributed (bottom).

The shallow network architecture facilitated tracing back the relevance of each data point in the EEG to a given prediction. We observed an uneven distribution of relevance over time when we worked with raw EEG data: the relevance was attributed mostly to artifactual signals during the electrocardiac QRS complexes, and not to neural waveforms. There are two possible explanations why the network focuses on the EEG‐visible heart activity: There could be sex differences (i) directly in the source of the electrocardiac activity, or (ii) in the volume conducting body that lead to differences in the electrical potentials at the sensors. Electrocardiac sex differences are well known (Macfarlane, 2018; Simonson et al., 1960) and have been detected with deep learning from 12‐channel ECG (Attia et al., 2019). However, our network architecture—when adapted to the single ECG channel instead of the 21‐channel EEG input—was unable to detect the sex from it. Detecting the sex from the heart signals within the EEG curves, but not from the single‐channel ECG, could be explained by differences emerging when the signals propagate from the heart to the scalp. ICA‐based artifact rejection strongly mitigated the electrocardiac artifacts in the EEG and led to evenly distributed relevance attribution over the time series, but also to a loss of 4% accuracy.

The body of literature on EEG sex differences agrees on frequency‐specific differences in normal (Cave & Barry, 2021; Friedl & Vogel, 1979b; Smith, 1952) and abnormal EEGs (Vogel & Götze, 1962), particularly in the β‐band. In this study, however, none of the resulting frequency‐specific accuracies stood out substantially. The differences between the frequency bands were either insignificant or small and could be related to different signal‐to‐noise ratios in the different frequency bands, rather than frequency‐specific sex differences. The discrete kernel grid might also impose a slight bias against higher frequencies. Cave and Barry (2021) found sex differences in EEG topographies. In agreement to that, sex detection with our CNN was possible even after the complete destruction of waveforms, frequencies, and connectivities by shuffling the time points while keeping the topographies in good order. Compared with the intact time series, the accuracy dropped only by ≈6%. We further investigated whether this accuracy gap should be attributed to actual time‐domain information or just to the fact that topographies are likely more robustly detectable from a series of samples, rather than just one point in time. After destroying the topographies by randomly shifting channel by channel forward or backward while keeping the channel‐wise waveforms and frequencies in good order, sex detection was no longer possible. This provides evidence for sex‐typical topographies. Again, different from the above‐mentioned studies, our segments were arbitrarily extracted from clinical recordings and exhibited a mix of paradigms with resting states, stimuli, and so forth. Sex differences in the waveforms or frequencies either could not be learned by our CNN or were not sufficiently present in the segments that we extracted from this heterogeneous dataset. Another explanation for why our network did not find differences in the waveforms or frequencies could be that the sex differences therefrom might have high variations of frequency, amplitude, and location (Friedl & Vogel, 1979b; Vogel & Götze, 1962), which is difficult to represent with simple convolutional kernels. Deeper neural networks might be able to better adapt to these nuances. In contrast to the studies that found sex differences in certain frequency bands under controlled conditions, our data from miscellaneous clinical scenarios exhibit a multitude of different conditions. Frequency‐specific sex differences might be better observed from data with a homogeneous paradigm.

The importance of topographies rather than waveforms might indicate that the differences arise from different propagation of the signals through the volume, rather than different neural activity. Furthermore, for neural sources, we would expect stronger differences between the accuracies achieved from the individual frequency bands. Together, this suggests anatomical differences as one cause of the different EEG signals.

Our results do not suggest the sex differences to be focused on a certain brain region. While the frontal channels had the highest single‐channel accuracies, the search for optimal additions of further channels yielded a central, temporal, parietal, and occipital channel.

The relevance attribution maps from males and females each showed correlations with the kernels from both sexes, indicating a substantial overlap in the EEG activity of males and females. This overlap is in agreement with previous reports on sex differences in the EEG (Friedl & Vogel, 1979b; Smith, 1952) and other brain‐related differences (Eliot et al., 2021; Weis et al., 2020). The studies by van Putten et al. (2018) using deep learning, Maciejewska and Froelich (2021) using naive Bayes and random forests, and this work using a shallow CNN all achieved ≈80% accuracy. These recurring 80% may indicate a limit, potentially from an overlap of the underlying properties causing the sex differences.

The detectability of the sex from EEG, which records the electrical potentials on the scalp, by no means provides evidence for dimorphic male and female brain *function*. The undisputed sex differences in brain size and the brain‐size‐related gray matter to white matter ratio (Eliot et al., 2021) influence how the brain signals propagate through the tissue toward the EEG sensors (Haueisen et al., 2002). After all, the differences are not necessarily rooted in the central nervous system, but could as well originate from the heart, eyes, muscular activity, movement patterns, and from the volume conducting anatomy, through which all of the electrophysiological signals must propagate (Hari & Puce, 2017). With the typically larger datasets in deep learning‐based studies, artifacts from such non‐neural sources might be more common than in conventional EEG studies because the individual recordings are treated less carefully. The extensive review by Craik et al. (2019) on deep learning for EEG lists various studies for disease detection where artifacts were left in during analysis.

A notable difference between van Putten et al.'s study and ours is the demographics: We used data from clinical patients and almost all of them matched the exclusion criteria in van Putten et al.'s study. We included only recordings that were labeled as *normal* by trained neurologists, but more than half of the patients in our study had epilepsy, affective disorders, or headache, and more than half received medication that has known effects on the EEG, such as antiepileptics, antidepressants, and benzodiazepines (Duncan, 1987; Haueisen et al., 2000; Meador et al., 1993). Table S3 presents the patient demographics, medical history, and reported medication. Sex‐related imbalances in the documented diseases and medications were not observed to an extent that would allow the CNN to detect the sex entirely based on diseases predominated by one sex. Despite the markedly different demographics and a much smaller network than in the study from van Putten et al. (ours: 6400 trainable parameters compared with theirs: 9 million), we received precisely the same accuracy (81%, balanced accuracy). The purpose of reducing our network to such low capacity was to create precedence: if our shallow CNN can detect the sex, the much deeper and therewith more capable networks that have been proposed for disease estimation can detect the sex as well. Most of the recently proposed architectures have millions of parameters (see the reviews by Roy et al. (2019) and Craik et al. (2019)).

## 5 CONCLUSION

We replicated neural network‐based sex detection from EEG. Our work introduces a method for relevance attribution for the case of a shallow neural network and presents the sex‐related patterns in the EEG data. None of the traditional frequency bands were particularly important for sex detection. Non‐neural sources, particularly from the heart, were found to inflict sex‐biased components to the EEG, but even after rejecting these artifacts, the sex remained highly detectable.

Our findings suggest that sex is easily detectable by practically any neural network for EEG analysis. For the many neurological and psychiatric diseases with unequal prevalences for males and females, sex is therefore a likely hidden confounder. Disease classifiers should therefore be analyzed for sex biases, for example by comparing if the detection performance for one sex is different for male‐ or female‐only trained models.

Researchers using large datasets should aim to maintain best practices of artifact rejection and employ explainability methods to identify illegitimate patterns, such as the heart‐related signals revealed in our study.

## CONFLICT OF INTEREST STATEMENT

The authors declare no potential conflict of interest.

## Supporting information


**Figure S1.** Average artifact load over time. At the beginning of a recording, the number of bad channels is typically increased, for example, due to increased movement or sensor readjustments. This graph shows that for the dataset used in this study, the average number of bad channels was stable after around 100 s. In our work, we always skipped the first 120 s.
**Figure S2.** Accuracy of the sex detection from different electroencephalogram (EEG) segment lengths. Markers denote the median value from five repeated experiments, and the error bars range from the best to the worst result. Preferably, the segments are as short as necessary. The influence of the segment length was small and flattened after a value of 4 s. Therefore, we chose 4 s as the segment length in this study.
**Figure S3.** Accuracy of the sex detection from different kernel matrix lengths and numbers. The kernel length is given in matrix elements, where one element is 4 ms long. The differences between the resulting accuracies at different parameters were below the standard deviation (3%) except for very few and very short kernels (upper left corner of the map). We chose a rather small number of kernels for better interpretability and to visualize them in a printable format (16 kernels with 19 samples = 76 ms length).
**Figure S4.** Spatio‐temporal kernels, trained on raw data without artifact removal. Some of the learned waveforms seemingly resemble neural oscillations. Still, the relevance attribution (Figures 3 and 4 in the main text) shows that the most relevant data points are associated with electrocardiac artifacts during the QRS complexes.
**Table S1.** Two‐tailed paired *t*‐test between the (imbalanced) accuracies of the models trained on different physiological bands of the ICA‐cleaned data. The table shows the pairwise *p*‐values.
**Table S2.** Two‐tailed paired *t*‐test between the (imbalanced) accuracies of the models trained on different physiological bands of the ICA‐cleaned and time‐shuffled data. The table shows the pairwise *p*‐values.
**Table S3.** Patient demographics of the separate evaluation set. The information was derived by a neurologist from the clinical reports in the TUH EEG corpus. The reports contain unstructured text that describes the patient, relevant history, medications, and clinical impression.

## Data Availability

The data analysis source code to replicate all results in this work is openly available in the Zenodo repository, https://doi.org/10.5281/zenodo.8098388. The Temple University Hospital Abnormal EEG Corpus (Lopez de Diego, 2017; Obeid and Picone, 2016) is available from www.nedcdata.org.
