# Supplementary material for: Sex‐related patterns in the electroencephalogram and their relevance in machine learning classifiers
Source: Hum Brain Mapp. 2023 Jul 17;44(14):4848–58. doi: 10.1002/hbm.26417 (PMC10472918; doi:10.1002/hbm.26417)
Supplement: Supplementary file 1 — Figure S1. Average artifact load over time. At the beginning of a recording, the number of bad channels is typically increased, for example, due to increased movement or sensor readjustments. This graph shows that for the dataset used in this study, the average number of bad channels was stable after around 100 s. In our work, we always skipped the first 120 s. Figure S2. Accuracy of the sex detection from different electroencephalogram (EEG) segment lengths. Markers denote the median value from five repeated experiments, and the error bars range from the best to the worst result. Preferably, the segments are as short as necessary. The influence of the segment length was small and flattened after a value of 4 s. Therefore, we chose 4 s as the segment length in this study. Figure S3. Accuracy of the sex detection from different kernel matrix lengths and numbers. The kernel length is given in matrix elements, where one element is 4 ms long. The differences between the resulting accuracies at different parameters were below the standard deviation (3%) except for very few and very short kernels (upper left corner of the map). We chose a rather small number of kernels for better interpretability and to visualize them in a printable format (16 kernels with 19 samples = 76 ms length). Figure S4. Spatio‐temporal kernels, trained on raw data without artifact removal. Some of the learned waveforms seemingly resemble neural oscillations. Still, the relevance attribution (Figures 3 and 4 in the main text) shows that the most relevant data points are associated with electrocardiac artifacts during the QRS complexes. Table S1. Two‐tailed paired t‐test between the (imbalanced) accuracies of the models trained on different physiological bands of the ICA‐cleaned data. The table shows the pairwise p‐values. Table S2. Two‐tailed paired t‐test between the (imbalanced) accuracies of the models trained on different physiological bands of the ICA‐cleaned and time‐shuffled data. The table [file HBM-44-4848-s001.pdf]

Sex-related patterns in the EEG and their relevance in machine learning classifiers: supplementary information

Thomas Jochmann[1,*], Marc S. Seibel[1], Elisabeth Jochmann[2], Sheraz Khan[3,4], Matti S. Hämäläinen[3,4,5], Jens Haueisen[1,2]

[1] *Department of Computer Science and Automation, Technische Universität Ilmenau, Ilmenau, Germany*
[2] *Department of Neurology, Jena University Hospital, Jena, Germany*
[3] *Athinoula A. Martinos Center for Biomedical Imaging, Massachusetts General Hospital, Charlestown, Massachusetts, USA*
[4] *Harvard Medical School, Boston, Massachusetts, USA*
[5] *Department of Neuroscience and Biomedical Engineering, School of Science, Aalto University, Espoo, Finland*

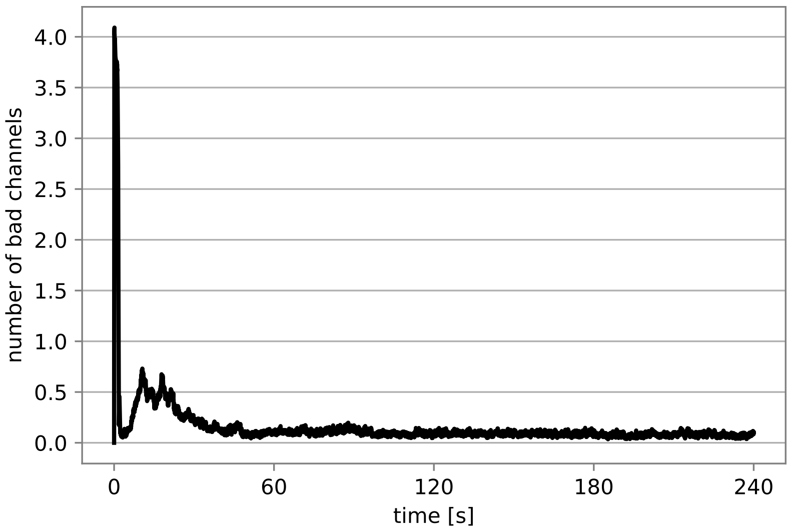

Figure S1: **Average artifact load over time.** At the beginning of a recording, the number of bad channels is typically increased, for example, due to increased movement or sensor readjustments. This graph shows that for the dataset used in this study, the average number of bad channels was stable after around 100 s. In our work, we always skipped the first 120 s.

*Corresponding author: Thomas Jochmann, Technische Universität Ilmenau, Institut für Biomedizinische Technik und Informatik, PF 10 05 65, 98684 Ilmenau, Germany

Email address: thomas.jochmann@tu-ilmenau.de (Thomas Jochmann)

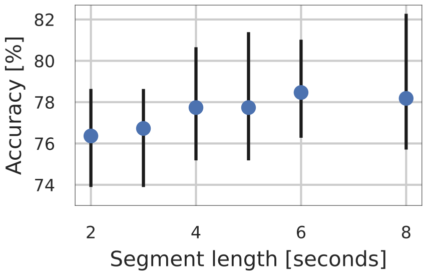

Figure S2: **Accuracy of the sex detection from different electroencephalogram (EEG) segment lengths.** Markers denote the median value from five repeated experiments, and the error bars range from the best to the worst result. Preferably, the segments are as short as necessary. The influence of the segment length was small and flattened after a value of 4 s. Therefore, we chose 4 seconds as the segment length in this study.

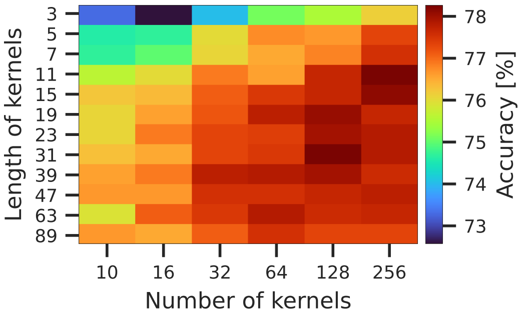

Figure S3: **Accuracy of the sex detection from different kernel matrix lengths and numbers.** The kernel length is given in matrix elements, where one element is 4 ms long. The differences between the resulting accuracies at different parameters were below the standard deviation (3%) except for very few and very short kernels (upper left corner of the map). We chose a rather small number of kernels for better interpretability and to visualize them in a printable format (16 kernels with 19 samples $\widehat{=}$ 76 ms length).

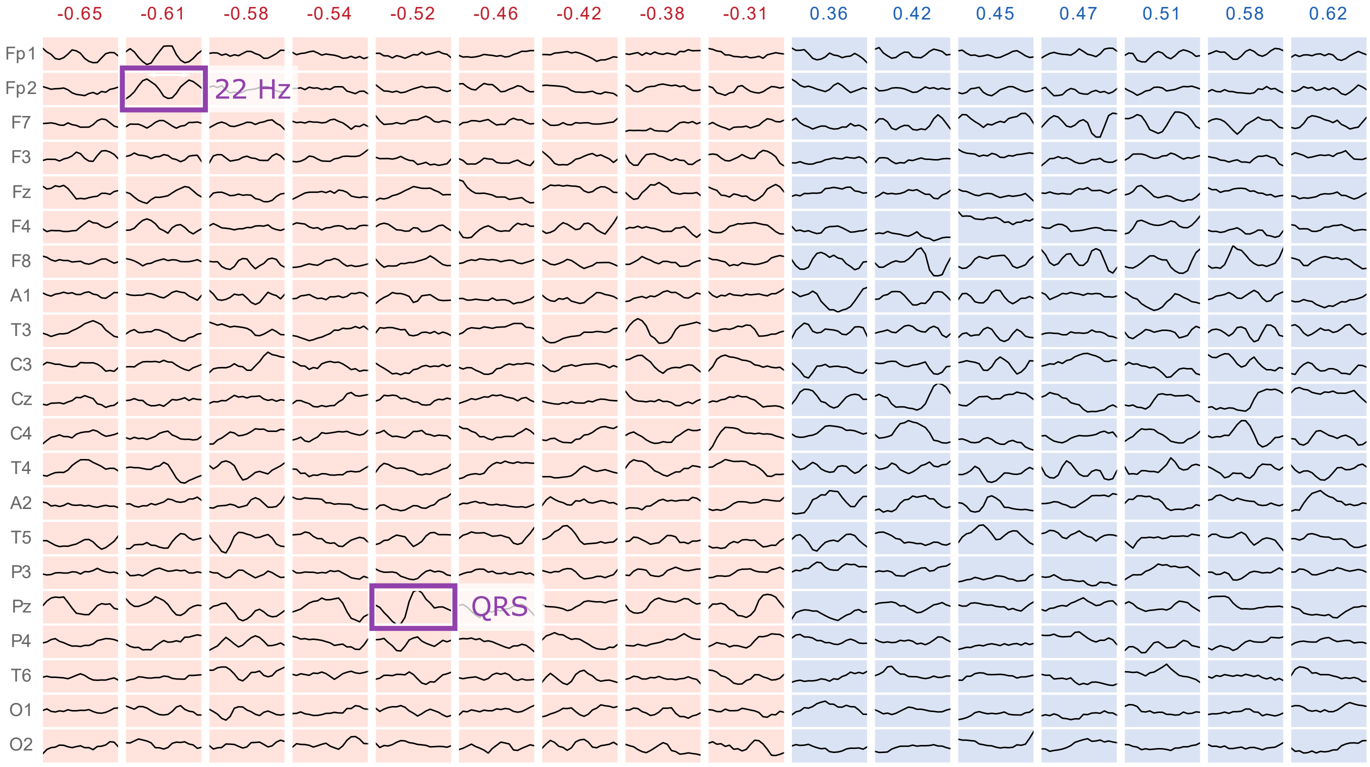

Figure S4: **Spatio-temporal kernels, trained on raw data without artifact removal.** Some of the learned waveforms seemingly resemble neural oscillations. Still, the relevance attribution (Figures 3 and 4 in the main text) shows that the most relevant data points are associated with electrocardiac artifacts during the QRS complexes.

Table S1: **Two-tailed paired t-test test between the (imbalanced) accuracies of the models trained on different physiological bands of the ICA-cleaned data.** The table shows the pairwise p-values.

	1 - 4 Hz (δ)	4 - 8 Hz (θ)	8 - 14 Hz (α)	14 - 30 Hz (β)	30 - 40 Hz (γ)
1- 40 Hz	0.077	0.21	0.80	$< .001$	$< .001$
1 - 4 Hz (δ)		0.62	0.045	$< .001$	$< .001$
4 - 8 Hz (θ)			0.12	$< .001$	$< .001$
8 - 14 Hz (α)				$< .001$	$< .001$
14 - 30 Hz (β)					0.03

Table S2: **Two-tailed paired t-test between the (imbalanced) accuracies of the models trained on different physiological bands of the ICA-cleaned *and time-shuffled* data.** The table shows the pairwise p-values.

	1 - 4 Hz (δ)	4 - 8 Hz (θ)	8 - 14 Hz (α)	14 - 30 Hz (β)	30 - 40 Hz (γ)
1- 40 Hz	$< .001$	0.015	$< .001$	$< .001$	0.21
1 - 4 Hz (δ)		$< .001$	0.44	0.54	$< .001$
4 - 8 Hz (θ)			$< .001$	0.005	0.12
8 - 14 Hz (α)				0.29	$< .001$
14 - 30 Hz (β)					$< .001$

Table S3: **Patient demographics of the separate evaluation set.** The information was derived by a neurologist from the clinical reports in the TUH EEG corpus. The reports contain unstructured text that describes the patient, relevant history, medications, and clinical impression.

	total	male	female
Demographics			
count	142 (100%)	61 (43%)	81 (57%)
age	45±17 y.	43±15 y.	46±18 y.
Medical history			
epilepsy	51 (36%)	24	27
affective disorders	12 (8%)	1	11
headache	18 (13%)	7	11
Medication			
no reported medication	30 (21%)	18	12
Antiepileptics	37 (26%)	14	23
Antidepressants	7 (5%)	2	5
Benzodiazepines	18 (13%)	5	13
